# The Cytosolic DNA-Sensing cGAS-STING Pathway in Liver Diseases

**DOI:** 10.3389/fcell.2021.717610

**Published:** 2021-07-27

**Authors:** Zhilei Wang, Nian Chen, Zhiyong Li, Guang Xu, Xiaoyan Zhan, Jianyuan Tang, Xiaohe Xiao, Zhaofang Bai

**Affiliations:** ^1^TCM Regulating Metabolic Diseases Key Laboratory of Sichuan Province, Hospital of Chengdu University of Traditional Chinese Medicine, Chengdu, China; ^2^Department of Liver Diseases, The Fifth Medical Centre, Chinese PLA General Hospital, Beijing, China; ^3^State Key Laboratory of Southwestern Chinese Medicine Resources, School of Pharmacy, Chengdu University of Traditional Chinese Medicine, Chengdu, China; ^4^China Military Institute of Chinese Materia, The Fifth Medical Centre, Chinese PLA General Hospital, Beijing, China

**Keywords:** DNA-sensing, cyclic GMP-AMP synthase, stimulator of interferon genes, liver diseases, cyclic guanosine monophosphate-adenosine monophosphate

## Abstract

Inflammation is regulated by the host and is a protective response activated by the evolutionarily conserved immune system in response to harmful stimuli, such as dead cells or pathogens. cGAS-STING pathway is a vital natural sensor of host immunity that can defend various tissues and organs against pathogenic infection, metabolic syndrome, cellular stress and cancer metastasis. The potential impact of cGAS-STING pathway in hepatic ischemia reperfusion (I/R) injury, alcoholic/non-alcoholic steatohepatitis (ASH), hepatic B virus infection, and other liver diseases has recently attracted widespread attention. In this review, the relationship between cGAS-STING pathway and the pathophysiological mechanisms and progression of liver diseases is summarized. Additionally, we discuss various pharmacological agonists and antagonists of cGAS-STING signaling as novel therapeutics for the treatment of liver diseases. A detailed understanding of mechanisms and biology of this pathway will lay a foundation for the development and clinical application of therapies for related liver diseases.

## Introduction

As a first line of defense, the innate immune system identifies extracellular or intracellular pathogenic-associated molecular patterns (PAMPs) and damage-associated molecular patterns (DAMPs) through pattern recognition receptors (PRRs) (Tang et al., 2012; Gong et al., 2020; Zindel and Kubes, 2020). Transmembrane receptors and cytoplasmic receptors, including NOD-like receptors (NLRs), toll-like receptors (TLRs), C-type lectin receptors (CLRs), and RIG-I-like receptors (RLRs), are the main susceptor for pathogens or danger signals (Kim et al., 2016; Zhu et al., 2018; McKernan, 2020). These receptors’ signal domains are located in the cytoplasm and initiate a signal cascade reaction to produce a series of gene products related to immune and inflammatory responses after binding with ligands. When a pathogenic organism enters and replicates within the cell, cytoplasmic sensors that activate different signaling pathways are activated. For example, NLRs can activate inflammasomes and trigger downstream signaling pathways by identifying various microbial molecules, toxins and damaged cells, and induce inflammatory responses and interleukin 1β (IL-1β) and IL-18 production (Davis et al., 2011; Man and Kanneganti, 2015). Cytoplasmic RLRs trigger signal cascades by recognizing virus and host-derived RNAs in the cytoplasm, leading to the secretion of type I interferon and inflammatory cytokines (Kawai and Akira, 2008; Kato et al., 2011; Kato and Fujita, 2015). Cyclic guanosine monophosphate (GMP)-adenosine monophosphate (AMP) synthase (cGAS) is a cellular DNA receptor that primarily recognizes viruses, bacteria and endogenous double-stranded DNA (dsDNA) irrespective of the sequence to activate innate immune responses and induce interferon expression and pro-inflammatory cytokines (Hopfner and Hornung, 2020; Wan et al., 2020).

Hepatitis viruses, especially hepatitis B virus (HBV), are DNA viruses and the leading cause of chronic viral hepatitis. In addition, the function of the liver is highly dependent on mitochondria for the production of ATP for biosynthesis and detoxification effects (Rui, 2014). There is growing evidence that most liver diseases are characterized by severe mitochondrial dysfunction (Paradies et al., 2014; Mansouri et al., 2018), which is manifested not only by the destruction and depletion of mitochondrial DNA (mtDNA), but also by the release of mtDNA (Malik and Czajka, 2013; Bose and Beal, 2016; Sharma et al., 2019). Moreover, the occurrence of liver diseases may induce necrosis, apoptosis, pyroptosis and other death processes in liver parenchymal and non-parenchymal cells (NPCs), resulting in DNA damage (Najimi et al., 2009; Chen et al., 2018; Xu et al., 2021). cGAS has now also been found to interact with various endogenous self-DNAs, including nuclear and mitochondrial cytoplasmic DNA, cytoplasmic micronucleus DNA, and nuclear chromatin. The inhibitory or activating effect of the cGAS-stimulator of interferon genes (STING) pathway plays a vital role in the occurrence and development of non-alcoholic fatty liver disease (NAFLD), alcoholic liver disease (ALD), viral hepatitis, liver fibrosis, liver cancer and other liver diseases. In this review, we discuss cGAS-STING signaling in liver diseases, and the profound implications of this pathway for novel therapeutic approaches against liver diseases.

## The cGas-Sting Signaling Pathway

### DNA Sensing cGAS

In 2013, James Chen used liquid chromatography-mass spectrometry to discover cyclic guanosine monophosphate-adenosine monophosphate (cGAMP), a substance that can activate cells to produce type I IFN (Wu et al., 2013). cGAMP functions as an endogenous second messenger and binds to STING to induce type I IFN in response to cytosolic DNA (Wu et al., 2013; Kato et al., 2017). Then, through protein purification and mass spectrometry, a nucleotidyl transferase family cGAMP synthase (cGAS) was identified (Sun et al., 2013). It was found that DNA in the cytoplasm can activate cGAS, and cGAMP catalyzed by cGAS can activate downstream transcription factor interferon regulatory factor 3 (IRF3) and induce the expression of STING dependent IFNβ (Cai et al., 2014; Chen Q. et al., 2016). Functional studies of cGAS mutants have shown that the conserved active sites G212, S213, E225, and D227 are necessary for cGAS activity, and mutations C396A and C397A decrease cGAS activity in humans (Civril et al., 2013). In addition to exogenous DNA, endogenous mtDNA can also be recognized by cGAS after entering the cytoplasm and triggering the immune response (Aarreberg et al., 2019; Maekawa et al., 2019; Huang et al., 2020). mtDNA is a circular double strand of about 17 Kbp, which can encode various enzymes and related ribosomal RNA and transport RNA required for oxidative phosphorylation, and has characteristics similar to prokaryotic nucleic acids (Banoth and Cassel, 2018). Under normal circumstances, mtDNA exists in the mitochondrial matrix, but it can be released into the cytosol or into circulation during cellular stress or mitochondrial injury, and then bind with DNA receptor cGAS to activate the cGAS-STING signaling pathway and enhance type I IFN responses (West et al., 2015).

### cGAS-STING Activation

**FIGURE 1:**
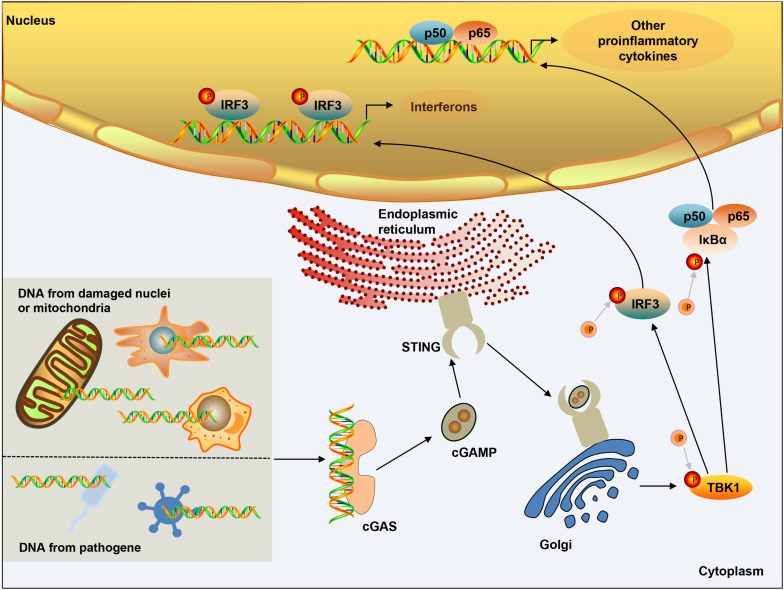
cGAS-STING signaling activation. DNA from microbial pathogens, damaged nuclei, or mitochondria binds and activates cGAS, and catalyzes the synthesis of cGAMP. cGAMP functions as a second messenger and binds to the ER-membrane adaptor STING and induces a conformational change that presumably results in the activation of STING. STING traffics from the ER to an ER-Golgi intermediate compartment and the Golgi apparatus, and then activates TBK1. TBK1 phosphorylates the transcription factor IRF3. STING also activates NF-kB, which enters the nucleus, where it functions together with IRF3 to induce the expression of type I IFNs and many other genes that mediate immune and inflammatory responses.

DNA in the cytoplasm is a powerful activator of the type I interferon response. In the normal physiological state of the body, DNA exists in the nucleus and mitochondria, and nucleases in the cytoplasm and endolysosomal compartments can quickly degrade DNA. However, when the organism is infected, the increase in the amount of intracellular DNA can be detected, which is closely related to the activation of the cGAS pathway. cGAS is a member of the nucleotide transferase (NTase) enzyme family, consisting of one NTase domain and two major DNA-binding domains (Sun et al., 2013). In the normal physiological state, DNA exists in the nucleus and mitochondria, and nucleases in the cytoplasm and endolysosomal compartments can quickly degrade DNA. Cytosolic DNA is required for cGAS activation, therefore, in the absence of DNA, cGAS is maintained an autoinhibited state (Civril et al., 2013; Li X. et al., 2013; Zhang X. et al., 2014). Once bound to DNA, cGAS can form a 2:2 complex that induces conformational changes at the active site and further catalyzes the synthesis of cGAMP from ATP and GTP (Ablasser et al., 2013a; Gao et al., 2013; Li X. D. et al., 2013). As a second messenger, cGAMP binds to the adaptor protein STING on the endoplasmic reticulum (ER) membrane, and induces a conformational change that may lead to the activation of STING (Shang et al., 2019). STING is transferred from the ER to the intermediate compartment of the ER-Golgi and Golgi apparatus, and then activates TANK-binding kinase 1 (TBK1) (Zhao et al., 2019). TBK1 then undergoes phosphorylation of itself and STING, followed by the IRF3 transcription factor. IRF3 dimerizes and enters the nucleus, triggering the production of type I interferon (Zhang et al., 2019). STING can also activate IKK kinase, then phosphorylate the IkB family of inhibitors of the transcription factor NF-kB (Ishikawa and Barber, 2008), and finally induce inflammatory cytokines such as tumor necrosis factor (TNF) and IL-6 (Figure 1). The cGAS-STING pathway activation has been shown to activate canonical and – in cancer cells – non-canonical nuclear factor kB (NF-kB), mitogen-activated protein (MAP) kinases, and signal transducer and activator of transcription (STAT) transcription factors (McWhirter et al., 2009; Bakhoum et al., 2018). In addition, after the activation of cGAS-STING signaling pathway can induce apoptosis (Zierhut et al., 2019; Li et al., 2021; Reinert et al., 2021), pyroptosis (Gaidt et al., 2017; Swanson et al., 2017), necroptosis (Schock et al., 2017), autophagy (Gui et al., 2019; Liu D. et al., 2019), and cell senescence (Yu et al., 2015; Glück and Guey, 2017; Yang et al., 2017).

### Regulation of the cGAS-STING Pathway of Cytosolic DNA Sensing

#### Regulation of cGAS Activation

**FIGURE 2:**
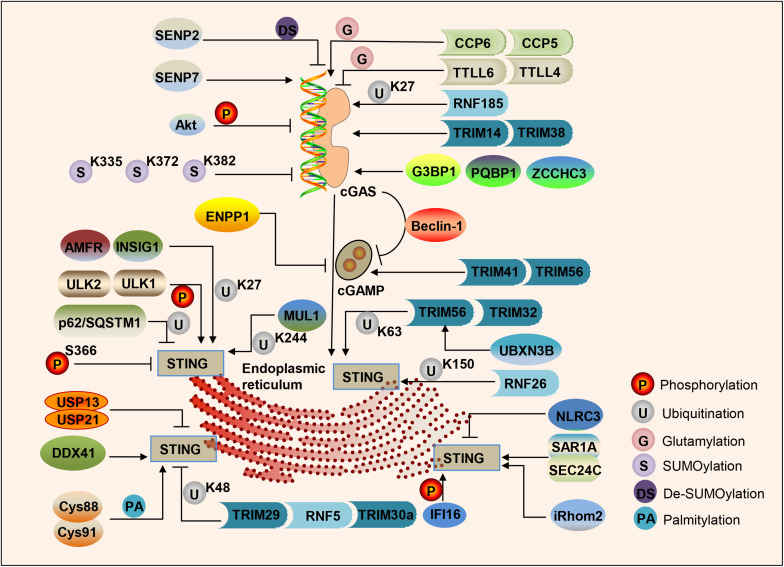
Regulation of the cGAS-STING pathway of cytosolic DNA sensing. cGAS, cGAMP and STING are dynamically regulated by various modifications in response to DNA attacks, such as phosphorylation, ubiquitination, SUMOylation, glutamylation, palmitoylation, and the protein–protein binding in cells.

cGAS binds to the sugar-phosphate backbone of dsDNA independently of the DNA sequence (Li X. et al., 2013; Zhang X. et al., 2014). Oxidized DNA is more resistant to cellular nucleases, leading to stronger induction of interferon (Gehrke et al., 2013). Subsequent research has found that single-stranded DNA that can form an internal double-stranded structure can induce the activation of cGAS (Herzner et al., 2015). Various studies have shown that the post-translational modification of cGAS can adjust its enzymatic activity. The ER ubiquitin ligase RNF185 specifically catalyzes the polyubiquitination of cGAS’s K27 linkage and promotes cGAS activity (Wang et al., 2017). Ser305 in human cGAS (Ser291 in mouse cGAS) can be phosphorylated by the kinase Akt, and this phosphorylation can inhibit the activity of cGAS (Seo et al., 2015). Type I IFN induces TRIM14 to accelerate cGAS stabilization by recruiting USP14 to cleave the cGAS ubiquitin chain at lysine 414 of cGAS (Chen M. et al., 2016). cGAS can be polyglutamylated by the enzyme TTLL6 and monoglutamylated by TTLL4, and those modifications inhibit the activity of cGAS (Xia et al., 2016). The carboxypeptidases CCP6 and CCP5 can reverse this modification and activate cGAS activity (Xia et al., 2016). Small ubiquitin-like modifiers (SUMO) are coupled to lysine residues 335, 372, and 382 of cGAS to inhibit cGAS-DNA binding, oligomerization, and nucleotidyl transferase activity. The ubiquitin ligase TRIM38 can target cGAS for SUMOylation, which prevents its polyubiquitination and degradation and increases its stability (Hu et al., 2016). In contrast, cGAS is De-SUMOylated through SENP2 and then degraded through proteasome pathways (Hu et al., 2016). Similarly, SENP7 reverses this inhibition by catalyzing the De-SUMOlation of cGAS and enhances cGAS activation (Cui et al., 2017; Figure 2).

In addition to post-translational modification processes such as (De)ubiquitination, (De)glutamination, and (De)SUMOylation, protein–protein binding can also regulate the activity of cGAS. G3BP1 binds to the N-terminus of cGAS, increases DNA-binding affinity and primes cGAS for effective activation (Liu Z. S. et al., 2019). PQBP1 binds to cGAS via the PQBP1 WW domain and enhances cGAMP production (Yoh et al., 2015). ZCCHC3 directly binds to dsDNA to enhance the binding of cGAS and dsDNA (Lian et al., 2018). In addition, cGAS directly interacts with Beclin-1 autophagy protein, leading to inhibition of the synthesis of cGAMP, enhancing autophagy-mediated DNA degradation of cytoplasmic pathogens, and preventing excessive activation of cGAS and continuous immune stimulation (Liang et al., 2014; Figure 2).

#### Regulation of cGAMP Synthesis and Production

As a second messenger, the amount of cGAMP is regulated by its synthesis and decay rate. The extracellular enzyme ecto-nucleotide pyrophosphatase/phosphodiesterase (ENPP1) can degrade 2′3′-cGAMP with high specificity (Li et al., 2014). cGAMP can transfer between cells through gap junctions, allowing virus-infected cells to warn uninfected nearby cells to activate the interferon pathway to fight infection (Ablasser et al., 2013b), which is especially crucial for generating immune defenses in virus-infected cells. The E3 ubiquitin ligases TRIM41 and TRIM56 actively regulate cGAS-mediated cGAMP synthesis by promoting the monoubiquitination of cGAS (Liu et al., 2018; Seo et al., 2018; Figure 2).

#### Regulation of STING by Post-translational Modifications

TANK-binding kinase 1 phosphorylates several serine and threonine residues of STING, including Ser366. This phosphorylation of STING plays a crucial role in the subsequent phosphorylation of IRF3 by TBK1 (Liu S. et al., 2015). Phosphorylation of S366 can inhibit STING function, and UNC-51-like kinases ULK1 and 2 are responsible for phosphorylating STING on S366 (Konno et al., 2013). STING degradation can be mediated by p62/SQSTM1, which is phosphorylated by TBK1, leading to ubiquitination of STING to autophagosomes (Prabakaran et al., 2018). An E3 ubiquitin ligase RNF26 can promote K11-linked polyubiquitination of STING at lysine150 (Qin et al., 2014). Ubiquitin protein E3 ligases TRIM56 and TRIM32 can promote Lys63 polyubiquitination of STING, thereby enhancing its downstream pathway activation (Tsuchida et al., 2010; Zhang et al., 2012). The ER localization E3 ligase complex composed of AMFR and INSIG1 promotes K27-linked STING polyubiquitination, TBK1 recruitment, and interferon induction (Wang et al., 2014). UBXN3B interacts with STING and its E3 ligase TRIM56, and promotes STING’s ubiquitination, dimerization, trafficking, and subsequent recruitment and phosphorylation TBK1 (Yang et al., 2018). MUL1 can catalyze the K63-linked polyubiquitination of K224 on STING and facilitate STING trafficking (Ni and Konno, 2017). In contrast, E3 ligases TRIM29, RNF5, and TRIM30a can promote the K48 polyubiquitination of STING, which is degraded by proteasomes, finally leading to inhibition of the DNA-sensing pathway (Zhong et al., 2009; Wang et al., 2015; Xing et al., 2017). NLRC3 is related to both STING and TBK1, and prevents STING-TBK1 interaction and downstream type I interferon production (Zhang L. et al., 2014). CYLD partially accumulates with STING, and selectively interacts with its K48-linked polyubiquitin chains, specifically removing them from STING (Zhang et al., 2018). USP13 and USP21 negatively regulate the polyubiquitin chain from STING and prevent the recruitment of TBK1 to the signal complex, thereby negatively regulating the cellular antiviral response (Chen et al., 2017; Sun et al., 2017). Moreover, STING is palmitoylated at Cys88 and Cys91 of the Golgi apparatus, and the activation of STING can be blocked by palmitoylation inhibitor 2-bromopalmitate (2-BP) or by mutation of two cysteine residues (Cys88/91) to inhibit palmitoylation of STING (Mukai et al., 2016; Figure 2).

Furthermore, protein–protein binding also regulates STING trafficking and activation. SAR1A and SEC24C facilitate STING trafficking and are essential for STING signaling (Gui et al., 2019). A member of the DEXDc family of helicases, DDX41, binds to DNA and STING and localizes in the cytoplasm together with STING (Zhang et al., 2011; Parvatiyar et al., 2012). Interferon-γ-induced protein 16 (IFI16) interacts with STING to promote STING phosphorylation and translocation (Almine et al., 2017). In addition, IFI16 promotes the recruitment and activation of TBK1 in the STING complex (Jønsson et al., 2017). Inactive rhomboid protein 2 (iRhom2) recruits the translocon-related protein TRAPβ to the STING complex to promote the trafficking of STING. iRhom2 also recruits the deubiquitinating enzyme EIF3S5 to maintain the stability of STING by removing its K48-linked polyubiquitin chain (Luo et al., 2016). An NLR protein NLRX1 blocks the interaction of DNA-sensing adaptor STING with TBK1 (Guo et al., 2016). The Ca^2+^ sensor stromal interaction molecule 1 (STIM1) regulates the type I IFN response by retaining the adaptor protein STING at the ER (Srikanth et al., 2019; Figure 2).

In addition, the cGAS-STING signaling pathway is also regulated by various intracellular mechanisms. Rongvaux et al. found that activation of pro-apoptotic Bcl-2 family members Bak and Bax, which are located on the mitochondrial membrane, can trigger the release of mtDNA from mitochondria into the cytoplasm and activate the cGAS-STING signaling pathway (Rongvaux et al., 2014). However, the apoptosis-related caspase cascade proteins caspase-9, Apaf-1, or caspase-3/7 can inhibit Bak/Bax-mediated cGAS pathway activation and mtDNA-induced innate immune responses (White et al., 2014). Recently, Banerjee et al. found that Gasdermin D can inhibit the type I IFN response mediated by cGAS in macrophages. In addition, the negative regulation of Gasdermin D on IFN-β is independent of pyroptosis and inflammasome products IL-1β and IL-18 (Banerjee et al., 2018).

## cGas-Sting Pathway in Liver Diseases

### Hepatic I/R Injury

Liver ischemia reperfusion (I/R) is a complex process that can cause severe liver injury and even multiple organ dysfunction. During ischemia, the complex interaction between ER stress, oxidative stress, and inflammation leads to a large amount of hepatocyte loss during reperfusion, as well as a large number of cell death responses resulting in necrosis and apoptosis. The hepatotoxicity of I/R is caused by complex interactions between hepatocytes, tissue-resident immune cells, and immune cells recruited to the injury site. In this case, ER stress response, lipid signaling, and DAMPs released by dead hepatocytes play a major role in liver I/R (Brenner et al., 2013).

Enhanced protein levels of p-STING and p-TBK1 are observed in aged Kupffer cells (KCs) post-I/R and mtDNA stimulation, and aging facilitates STING overactivation, resulting in increased proinflammatory cytokines/chemokines production of macrophages, which ultimately aggravates liver I/R injury (Zhong et al., 2020). Targeting STING may be a key strategy for the treatment of liver I/R. STING siRNA abrogates the detrimental role of aging in aggravating liver I/R injury and intrahepatic inflammation. A specific inhibitor of STING, C-176, inhibits immoderate secretion of proinflammatory cytokines/chemokines in mtDNA-stimulated aged mice bone marrow-derived macrophages (Zhong et al., 2020). MicroRNA (miR)-24-3p downregulates of STING signaling and reduces expression of p-IRF3, serum inflammatory factors, and aminotransferase levels in liver I/R mice (Shen et al., 2020). In addition, distant organs (lung and liver) suffer severely by intestinal I/R injury, including increased levels of IL-6, IFN-β, and histopathological scores (Wu et al., 2021). However, STING^–/–^ ameliorates intestinal I/R-induced distant organ damage. This harmful effect relies on excessive lipid peroxidation, which has been confirmed by the levels of 4-hydroxynonenal and the malondialdehyde (Wu et al., 2021).

However, one study has also shown that I/R is not associated with activation of the STING pathway. In a non-lethal model of segmental (70%) hepatic warm I/R, cGAS knockout (cGAS^–/–^) mice have significantly increased serum ALT levels and areas of necrosis in liver sections compared with wild-type (WT) mice (Lei and Deng, 2018). Surprisingly, STING deficiency (STING^gt/gt^) does not phenocopy cGAS^–/–^ mice, suggesting a protective effect of cGAS in liver I/R independent of STING (Lei and Deng, 2018). Moreover, cGAS^–/–^ mice have increased cell death and decreased autophagy induction as well as increased apoptosis under hypoxic conditions, which may be due to increased mitochondrial dysfunction and subsequent increased oxidative stress damage (Lei and Deng, 2018). Therefore, whether the cGAS-STING signaling pathway plays a role in liver I/R still needs to be further explored and studied.

### Alcoholic Liver Disease

Alcoholic liver disease is the most common type of chronic liver disease worldwide including fatty liver, alcoholic hepatitis (AH), and cirrhosis with complications. ALD can gradually develop from alcoholic fatty liver (AFL) to alcoholic steatohepatitis (ASH), and is characterized by liver inflammation. Chronic ASH can eventually lead to fibrosis and cirrhosis, and in some cases even hepatocellular carcinoma (HCC). At present, fundamental treatments for ALD include abstinence, nutritional support, corticosteroids, and anti-TNF antibodies (Gao and Bataller, 2011; Louvet and Mathurin, 2015).

A few studies have shown that targeting the cGAS-STING pathway is a therapeutic strategy for ALD and is expected to play a vital role in its treatment. The expression of IRF3 and its related genes (cGAS, TBK1, IKKε, and STING) is positively correlated with the severity of ALD. One study has shown that pathogen DNA, produced by excessive drinking of alcohol, contributes to IRF3 activation in hepatic cells (Wang et al., 2011). Alcohol-fed mice have increased expression of cGAS-STING in the liver, while mice deficient in cGAS and IRF3 genes show a protective effect against ALD (Luther et al., 2020). The destruction of the predominant hepatic gap junction Connexin 32 (Cx32) in ALD impairs the expression of IRF3-stimulated genes, leading to reduced liver damage (Luther et al., 2020). Ethanol induces ER stress and triggers the association of IRF3 with the ER adaptor STING, as well as subsequent p-IRF3. Activated IRF3 is related to the proapoptotic molecule Bax and promotes hepatocyte apoptosis. Deficiency of STING inhibits the phosphorylation of IRF3 under ethanol or ER stress, and deletion of IRF3 prevents hepatocyte apoptosis (Petrasek et al., 2013). In summary, cGAS-STING is considered to be a key factor in the onset of ALD and a potential therapeutic target.

### Non-alcoholic Fatty Liver Disease and Non-alcoholic Steatohepatitis

Non-alcoholic fatty liver disease is one of the most important causes of liver disease worldwide, and may become the main cause of end-stage liver disease in the next few decades (Byrne and Targher, 2015; Younossi et al., 2018). NAFLD can occur in both adults and children, and it has a variable course but can lead to cirrhosis and liver cancer (Friedman et al., 2018). In addition, studies have shown that the clinical burden of NAFLD is not limited to liver-related morbidity and mortality, and growing evidence shows that NAFLD is a multi-system disease that affects extrahepatic organs and regulatory pathways (Byrne and Targher, 2015; Rinella and Sanyal, 2016). Non-alcoholic steatohepatitis (NASH) is considered to be a progressive form of NAFLD, characterized by liver steatosis, inflammation, hepatocellular damage, and varying degrees of fibrosis (Schuster et al., 2018). These causes of liver inflammation may come from outside the liver (such as adipose tissue or intestines) (Gäbele et al., 2011; Bijnen et al., 2018), or from inside organs (due to lipotoxicity, innate immune responses, cell death pathways, mitochondrial dysfunction, and ER stress) (Gao et al., 2018; Peng et al., 2018; Yu et al., 2019; Li et al., 2020), both of which contribute to the development of NAFLD/NASH. Therefore, innovative potential NAFLD/NASH therapies include four main pathways (Sumida and Yoneda, 2018): targeting liver fat accumulation; targeting oxidative stress, inflammation, and apoptosis; targeting intestinal microbiomes and metabolic endotoxemia; and targeting hepatic fibrosis. The contribution of cGAS-STING activation in NAFLD/NASH has therefore received substantial interest.

In clinical NAFLD/NASH patients, higher levels of STING in macrophages have been reported. The protein level of STING in non-parenchymal liver cells, including macrophages/KCs and endothelial cells, in liver tissues of NAFLD patients is higher than that in non-NAFLD patients (Luo et al., 2018). The number of STING + cells in the livers of NASH patients is increased with the severity of inflammation and the fibrosis stage (Wang et al., 2020). Compared with controls, the numbers of STING+/CCR2+ and STING+/S100A9+ macrophages in the livers of NASH patients are increased significantly, and they are positively correlated with the liver inflammation grade and fibrosis stage (Wang et al., 2020).

In animal models, cGAS-STING is observed to be involved in the formation of NAFLD/NASH. The expressions of STING and its downstream factor IRF3 are increased significantly in the livers of high-fat diet (HFD) mice (Li and Su, 2020). In HFD-fed mouse livers and free fatty acid (FFA)-induced LO2 cells, STING and IRF3 expression are up-regulated (Qiao et al., 2018). After HFD treatment, STING^gt^ mice and mice with destroyed myeloid cells (myeloid cell-specific STING disruption) show less liver steatosis, inflammation, and/or fibrosis, and after transplantation of bone marrow cells from mice in the control group to STING^gt^ mice, the severity of steatosis and inflammation after HFD can be restored (Luo et al., 2018). These results indicate that STING is specifically present in myeloid cells and can aggravate HFD-induced NAFLD. Moreover, the accumulation of gut microbial DNAs encapsulated with intestinal extracellular vesicles can activate cGAS-STING-mediated inflammatory signaling, which is pathogenic in the development of tissue inflammation and metabolic disorders (Luo et al., 2021).

Therefore, targeting cGAS-STING may be an effective way to treat NAFLD/NASH. In a NAFLD/NASH mouse model induced by methionine-choline deficient (MCD) and HFD, STING deficiency reduces liver steatosis, fibrosis and inflammation (Luo et al., 2018; Yu et al., 2019). Knock-out/down of STING or IRF3 can significantly reduce FFA-induced liver inflammation and apoptosis (Qiao et al., 2018). Meanwhile, HFD-fed mouse hepatocyte mtDNA induces the expression of TNF-α and IL-6 in cultured KCs, which can be attenuated by STING deficiency (Yu et al., 2019). Remdesivir (RDV) functions as an inhibitor of STING, inhibiting STING and IRF3 expression significantly in HFD-induced NAFLD (Li and Su, 2020). Chronic exposure to DMXAA, a mouse STING agonist, can cause liver steatosis and inflammation in WT mice, but not in STING-deficient mice (Yu et al., 2019). Through various mouse models and NAFLD/NASH patients, we have further confirmed that STING plays a harmful role in NAFLD/NASH. In summary, pharmacological inhibition of STING activation results in potent therapeutic effects in NAFLD/NASH, encouraging the development of novel therapeutics against this target.

### Hepatitis B Virus

Hepatitis B virus infection affects approximately 350 million people and is a major public health problem worldwide. HBV is the main cause of liver cirrhosis and HCC (Megahed and Zhou, 2020). The current non-therapeutic treatment is mainly nucleos(t)ide analogs (NAs), which can profoundly but not completely inhibit the DNA synthesis of viral reverse transcriptase (Pierra Rouviere et al., 2020). Increasing evidence indicates that HBV interacts with hepatocyte innate immune signaling pathways and inhibits innate immunity. Therefore, an in-depth understanding of the interaction between HBV virus and innate immunity is important for the development of new therapeutic strategies for the treatment of HBV infection.

Naked circle HBV DNA is sensed in a cGAS-dependent manner in liver cancer cell lines, immunocompetent myeloid cells and human primary hepatocytes (Verrier et al., 2018; Lauterbach-Rivière et al., 2020). When the cGAS-STING pathway is activated by dsDNA or cGAMP, whether in cell culture or in a mouse model, HBV replication is significantly inhibited (He et al., 2016). However, hepatocytes did not produce type 1 IFN upon foreign DNA stimulation or HBV infection, and mice lacking STING or cGAS show the same ability to control infection in an adenovirus-HBV model (Thomsen et al., 2016). Meanwhile, HBV infection suppresses the expression and function of cGAS in cell culture models and humanized mice, indicating that HBV exploits multiple strategies to circumvent the perception of cGAS and its effector pathways and antiviral activity (Verrier et al., 2018). Knockout of cGAS in human peripheral blood mononuclear cells leads to an increase in intracellular HBV DNA levels (He et al., 2016). Targeting of the cGAS-STING pathway may therefore be an effective strategy for treating HBV. Treatment of immortalized mouse liver cells that support HBV replication with cGAMP or small molecule pharmacological STING agonists significantly reduces viral DNA in a STING- and Janus kinase 1-dependent manner (Guo et al., 2017). DMXAA has been found to induce a strong cytokine response dominated by type I IFNs in macrophages. By reducing the number of cytoplasmic virus nucleocapsids, DMXAA effectively inhibits HBV replication in mouse hepatocytes (Guo et al., 2015). In addition, in an HBV hydrodynamic mouse model, intraperitoneal injection of DMXAA significantly induces the expression of IFN-stimulated genes and reduces HBV DNA replication intermediates in the mouse liver (Guo et al., 2015).

In terms of mechanistic research, the viral polymerase (Pol) of HBV can inhibit the activation of STING-stimulated IRF3 and the induction of IFN-β (Liu Y. et al., 2015). In addition, the reverse transcriptase and RNase H domains of Pol have been identified as the cause of this inhibition (Liu Y. et al., 2015). Moreover, Pol has been shown to be physically related to STING and significantly reduces the K63-linked polyubiquitination of STING through its reverse transcriptase domain, which provides a new mechanism for HBV to fight against the innate DNA sensing pathway (Liu Y. et al., 2015). The methylation frequency of the STING promoter in chronic hepatitis B patients is significantly higher, and the level of STING mRNA is significantly lower than that in healthy controls (Wu et al., 2018). Hypermethylation of the STING promoter and the resulting transcriptional inhibition of STING weaken the effect of STING in inhibiting HBV replication and reduce the effectiveness of antiviral therapy (Wu et al., 2018).

It is necessary to develop small-molecule human cGAS or STING agonists as immunotherapeutic drugs for the treatment of chronic hepatitis B. Daunorubicin (DNR), a topoisomerase II poison, triggers an endogenous cGAS-dependent innate immune response and subsequently inhibits the production of HBV in NKNT-3/NTCP cells (Imai et al., 2018). GV1001 inhibits HBV replication and hepatitis B surface antigen (HBsAg) secretion in a dose-dependent manner (Choi et al., 2020). GV1001, a telomerase-derived 16-mer peptide, promotes the release of oxidized DNA into the cytoplasm mediated by mtDNA stress and produces IFN-I-dependent anti-HBV effects through the STING-IRF3 axis (Choi et al., 2020). The anti-HBV effect of GV1001 is through translocation of extracellular heat shock proteins into the cytosol, leading to mtDNA stress mediated by the escape of phagosomes (Choi et al., 2020). Importantly, the specific introduction of STING expression in hepatocytes reconstructs the DNA sensing pathway, thereby improving the control of HBV *in vivo* (Thomsen et al., 2016). Therefore, activating the cGAS-STING pathway can induce the antiviral cytokine response to HBV.

### Liver Fibrosis

Liver fibrosis is the excessive accumulation of extracellular matrix proteins including collagen, and is common in most types of chronic liver disease (Bataller and Brenner, 2005). Advanced liver fibrosis can lead to cirrhosis, liver failure, and portal hypertension, and often requires liver transplantation (Zhou et al., 2014; Schuppan et al., 2018; Parola and Pinzani, 2019). Emerging anti-fibrosis therapies aim to inhibit the accumulation of fibroblasts and/or prevent the deposition of extracellular matrix proteins. Under stimulation by the main fibrogenic cytokine, transforming growth factor-β1 (TGF-β1), the expression of IRF3 in HSCs increases significantly (Ni et al., 2016). In addition, the LX-2 cell line transfected with IRF3-siRNA reduces the type I collagen (Col1a1) and α-smooth muscle actin (α-SMA) expression (Ni et al., 2016). Acute administration of CCl_4_ in WT mice results in early ER stress, IRF3 activation, and type I IFN induction, followed by hepatocyte apoptosis and liver damage, with liver fibrosis occurring after repeated administration of CCl_4_ (Iracheta-Vellve et al., 2016). In addition, Yong et al. (2021) found that activation of the cGAS/STING pathway caused by mitochondrial localization of TAR DNA-binding protein 43 (TDP-43) may be a pathogenic mechanism of CCl_4_-induced liver fibrosis. STING might therefore be a therapeutic target for liver fibrosis. IRF3 or STING deficiency can prevent acute or chronic CCl_4_ hepatocyte death and fibrosis; however, mice deficient in type I IFN receptor or TLR4 signal adaptor TRAM or TRIF are not protected from hepatocyte death and/or fibrosis, indicating that the pro-apoptotic effect of IRF3 is independent of TLR signaling in fibrosis (Iracheta-Vellve et al., 2016). IRF3 is associated with STING in the presence of ER stress and combines hepatocyte apoptosis with liver fibrosis, indicating that innate immune signals regulate the outcomes of liver fibrosis by regulating hepatocyte death in the liver (Iracheta-Vellve et al., 2016).

### Hepatocellular Carcinoma and Liver Metastasis

Liver cancer is a global challenge that threatens human health, and HCC accounts for approximately 90% of liver cancer cases and is its most common form. HBV and HCV infection are the main risk factors for the development of HCC. NASH associated with metabolic syndrome or diabetes is also a common risk factor in the West (Llovet et al., 2021). In HCC, immuno-oncology treatment methods have attracted much attention, including the use of checkpoint inhibitors, tyrosine kinase inhibitors or anti-VEGF therapy, and even a combination of two immunotherapies. However, we still need to optimize combination strategies to maximize the potential of these methods (Finn and Zhu, 2020).

In recent years, a large number of studies have reported a connection between the cGAS-STING signaling pathway and anti-tumor immunity, cancer progression, the tumor microenvironment, and pharmacological strategies for cancer treatment (Jiang et al., 2020; Kwon and Bakhoum, 2020; Wang, 2020). Prognostic biomarkers in HCC have been identified: the overexpressions of XRCC5, IRF3, TRIM21, STAT6, DDX41, TBK1, XRCC6, TREX1, PRKDC, and STING are significantly related to the clinical stage and pathological grades of HCC (Qi et al., 2020). Higher expressions of IFI16, STAT6, NLRC3, and STING mRNA are associated with a favorable overall survival rate (Qi et al., 2020). The cGAS-STING pathway is significantly related to the infiltration of B cells, CD4 + T cells, CD8 + T cells, macrophages, neutrophils, and dendritic cells in HCC (Qi et al., 2020). Compared with non-tumor tissues, the expression of STING protein in tumor tissues is significantly reduced, and the intensity of STING staining in HCC patients is negatively correlated with tumor size, tumor invasion tumor node metastasis (TNM) stage, and overall survival (Bu et al., 2016). The combined expression of STING and TNM staging has a better prognostic effect for overall survival in HCC patients (Bu et al., 2016). In the late stages of HCC induced by diethylnitrosamine, STING^gt/gt^ mice show larger tumors (Thomsen et al., 2020). Treatment of mice after HCC development with 3′3′-cAIMP, a cyclic dinucleotide STING agonist, effectively reduces the tumor size in cGAS^–/–^ mice, which cannot generate 2′3′-cGAMP (Thomsen et al., 2020). In addition, *Lactobacillus rhamnosus* GG induces cGAS/STING-dependent IFN-β production and improves the response to anti-programmed cell death 1 (PD-1) immunotherapy (Si et al., 2021). Therefore, regulation by STING pathway agonists may reverse the development of HCC and is expected to be an effective treatment strategy.

Liver metastasis remains a major obstacle to the successful treatment of malignant diseases, especially of gastrointestinal cancers and other malignant tumors, such as breast cancer and melanoma. The main cause of mortality from colorectal cancer is the development of liver metastases. Nanoencapsulation improves the half-life of encapsulated cGAMP by 40-fold, allowing for sufficient accumulation of cGAMP in tumors and activation of the STING pathway in the tumor microenvironment. In a B16-F10 melanoma model, nanoparticle delivery also changes the biodistribution profile, resulting in increased cGAMP accumulation and STING activation in the liver and spleen (Wehbe et al., 2020), which may be an important strategy for the treatment of liver metastases. E7766, a macrocyclic bridging STING agonist with a novel topology, shows extensive pan-genotypic activity in all major human STING variants. In a mouse liver metastasis model, E7766 has shown strong anti-tumor activity and a durable immune memory response (Kim et al., 2021).

In conclusion, STING agonists has an independent prognostic value and may be a potential target for HCC and liver metastasis immunotherapy. Clarifying the molecular mechanisms of STING agonists against HCC or liver metastases, searching for and screening out predictive markers that are suitable for STING agonist treatment, and selecting appropriate therapeutic doses to improve the effect of STING agonists on tumors and reduce their adverse effects are crucial for the clinical development of STING agonists. In addition, combined application with STING agonists can not only reduce the dosage and side effects of radiotherapy, chemotherapy, tumor vaccines, immune checkpoint inhibitors, chimeric antigen receptor T-cell (CAR-T) immunotherapy or other types of treatments, but also can synergistically enhance anti-tumor effects through different mechanisms.

### Other Liver Diseases

Other liver diseases have also been shown to be associated with the cGAS-STING pathway. In a two-thirds hepatectomy mouse model, the combined deficiency of MAVS and STING resulted in severely impaired hepatocyte proliferation and delayed the recovery of liver mass, indicating that the innate immune cytoplasmic nucleic acid sensor promotes liver regeneration (Schulze et al., 2018). Radiation causes dsDNA accumulation, and in NPCs, dsDNA rapidly activates the cGAS-STING pathway, leading to the production and release of IFN-I, accompanied by hepatocyte damage (Du et al., 2020). cGAS- or STING-deficient mice have alleviated radiation-induced liver steatosis and inflammation (Du et al., 2020). Moreover, clinically irradiated human liver tissue peri-HCC liver tissues show higher expression of STING and IFNβ than unirradiated tissue (Du et al., 2020). *Washingtonia robusta* leaves has been found to restore elevated liver index, ALT, albumin, cholesterol, and reactive oxygen species levels, and ameliorates the increase in STING expression in γ-radiation-induced oxidative hepatotoxicity (Selim et al., 2020). When acetaminophen is excessive, hepatocytes release a large amount of DNA, but these cells do not primarily sense DNA; however, liver NPCs quickly perceive this environmental interference and activate multiple DNA sensing pathways (Araujo and Antunes, 2018). Liver NPCs synthesize and release IFN-1, accompanied by hepatocyte necrosis. Ablation of IFN-1 in interferon α/β receptor (IFNAR^–/–^) mice delays APAP-mediated hepatic necrosis and inhibits the IFN-1 sensing pathway (Araujo and Antunes, 2018). In addition, although not a DNA virus, HCV virus NS4B protein inhibits STING and STING-induced IFN-β activation (Ding et al., 2013; Nitta et al., 2013; Yi et al., 2016). Interestingly, the expression of NS4B blocks the protein interaction between STING and Cardif, which is required for the activation of IFN-β(Nitta et al., 2013).

## Conclusion

As outlined in this review, the cGAS-STING pathway is a vital component of most acute and chronic liver diseases and contributes to liver I/R injury, ALD, NAFLD, NASH, HBV, fibrosis, and even HCC. Understanding the structural features and functions of cGAS-STING will provide new insights for the treatment of liver diseases based on this pathway through novel methods and agents. However, important questions remain in our understanding of how the liver regulates the cGAS-STING pathway to drive disease. Therefore, additional in-depth research on the cGAS-STING pathway will contribute to clinical treatments using cGAS-STING-related activators and inhibitors. In addition, when developing agonists or inhibitors that regulate the cGAS-STING signaling pathway, its potential adverse drug reactions are worthy of attention. For example, when using STING agonists for tumor immunotherapy, the treatment window and toxic side effects must be considered to avoid excessive activation of the STING pathway, which may cause severe inflammation, such as abnormally elevated cytokine levels (Gangadhar and Vonderheide, 2014; Cui et al., 2019). When using STING inhibitors to treat autoimmune diseases, excessive inhibition of the STING pathway may lead to a weakened immune system and significantly increase the risk of pathogen infection and tumor occurrence. The development of drugs that inhibit or promote the cGAS-STING signaling pathway has broad prospects for treating HCC, ALD, NASH, HBV, and other liver diseases, giving it a major role in basic immunology, tumor biology, and clinical treatment.

## Author Contributions

ZW, NC, and ZL: investigation, validation, visualization, and writing – original draft. XZ and GX: investigation, formal analysis, and data curation. JT, XX, and ZB: conceptualization, methodology, validation, resources, supervision, project administration, and funding acquisition. All authors contributed to the article and approved the submitted version.

## Conflict of Interest

The authors declare that the research was conducted in the absence of any commercial or financial relationships that could be construed as a potential conflict of interest.

## Publisher’s Note

All claims expressed in this article are solely those of the authors and do not necessarily represent those of their affiliated organizations, or those of the publisher, the editors and the reviewers. Any product that may be evaluated in this article, or claim that may be made by its manufacturer, is not guaranteed or endorsed by the publisher.
